# Development of an activation tagging system for maize

**DOI:** 10.1002/pld3.118

**Published:** 2019-02-21

**Authors:** John P. Davies, Vaka S. Reddy, Xing L. Liu, Avutu S. Reddy, William Michael Ainley, Otto Folkerts, Pradeep Marri, Ke Jiang, Douglas Ry Wagner

**Affiliations:** ^1^ Dow AgroSciences Indianapolis Indiana; ^2^ Present address: Molecular Microbiology School of Medicine Washington University in St Louis St Louis Missouri; ^3^ Present address: Genus PLC De Forest Wisconsin

**Keywords:** activation tagging, enhancer, insertional mutagenesis, transposable elements

## Abstract

Activation Tagging, distributing transcriptional enhancers throughout the genome to induce transcription of nearby genes, is a powerful tool for discovering the function of genes in plants. We have developed a transposable element system to distribute a novel activation tagging element throughout the genome of maize. The transposon system is built from the Enhancer/Suppressor (*En*/*Spm*) transposon system and uses an engineered seed color marker to show when the transposon excises. Both somatic and germinal excision events can be detected by the seed color. The activation tagging element is in a *Spm*‐derived non‐autonomous transposon and contains four copies of the *S*ugar*c*ane *B*acilliform *V*irus‐enhancer (*SCBV*‐enhancer) and the AAD1 selectable marker. We have demonstrated that the transposon can give rise to germinal excision events that can re‐integrate into non‐linked genomic locations. The transposon has remained active for three generations and events displaying high rates of germinal excision in the T2 generation have been identified. This system can generate large numbers of activation tagged maize lines that can be screened for agriculturally relevant phenotypes.

## 1 INTRODUCTION

Genetic analysis of mutants has proved to be an effective approach for determining the function of genes in numerous systems including plants. Insertional mutagenesis, where a DNA element integrates in genes, has been used in plant systems such as Arabidopsis, rice, and maize (Athma, Grotewold, & Peterson, 1992; Azpiroz‐Leehan & Feldmann, 1997; Bai, Singh, Pitt, Sweeney, & Brutnell, 2007; Errampalli et al., 1991; Sallaud et al., 2004; Walbot, 2000). These systems have been effective tools for gene discovery but most have the limitation of only being able to detect phenotypes caused by gene disruption that results in loss‐of‐function. Moreover, such mutations are generally recessive and only observed when in a homozygous state.

Activation tagging up‐regulates gene transcription by placing transcriptional enhancer elements or strong promoters near genes. Activation tagging systems have been developed for several plants including Arabidopsis, rice, and barley (Ayliffe, Pallotta, Langridge, & Pryor, 2007; Hsing et al., 2007; Jeong et al., 2002; Weigel et al., 2000). These systems have either used an array of 35S enhancers (Hsing et al., 2007; Jeong et al., 2002; Weigel et al., 2000) or a strong promoter (Ayliffe et al., 2007) to enhance transcription of genes near where the element inserts. Our previous study demonstrated the effectiveness of a sugarcane bacilliform virus enhancer (*SCBV* enhancer) in maize (Davies et al., 2014) and we have used this enhancer in this activation tagging platform.

Activation tagging populations have been generated in two ways; (a) by direct transformation of large numbers of plants and (b) by transforming plants with transposable elements engineered to distribute activation tagging elements. The engineered transposon systems have the advantage over direct transformation in that fewer transformation events need to be generated (Marsch‐Martinez, Greco, Van Arkel, Herrera‐Estrella, & Pereira, 2002; Qu, Desai, Wing, & Sundaresan, 2008; Schneider et al., 2005; Sundaresan, Qu, Desai, & Wing, 2008) and are especially useful in plants, such as maize, in which transformation is labor intensive.

Several engineered transposon systems have been used in plants (Walbot, 2000). In most of these systems there is a non‐autonomous transposon that is introduced into the plant along with a transposase that can excise the transposon and catalyze its integration at a new position; although some systems use an endogenous transposase. Most of the described systems take advantage of one of the maize transposon systems, *Ac*/*Ds*,* En*/*Spm* or *Mutator* (*Mu*). These transposon systems have different characteristics that influence the types of mutant populations that can be generated. For example, in *Ac*/*Ds* systems, the new sites of integration are generally near the site of the original insertion (Dooner & Belachew, 1989; Greenblatt, 1984) while in *En*/*Spm* and *Mu* systems the integration sites for the non‐autonomous elements are not usually linked to the original integration sites (Kumar, Wing, & Sundaresan, 2005; Lisch, Chomet, & Freeling, 1995). The transposase of the *En*/*Spm* system has been cloned and extensively characterized while the *Mu* transposase is less well characterized. Non‐autonomous *Mu* transposon systems rely on mutator lines that have an endogenous transposase that facilitates transposition.

We have developed a *Spm* transposon‐based activation tagging system for maize with the goal of generating a large activation tagged population. The goal of generating this population is to have tens of thousands of individual lines with insertions of the activation tagging element at different sites throughout the genome. To generate this population, the activation tagging transposon construct has been introduced into progenitor lines that serve as launch sites. Lines that undergo high rates of germ‐line excision of the transposable element have been identified. To easily distinguish between somatic and germ‐line excision events, a seed color system that supports the identification of germ‐line excision events was developed.

## 2 MATERIALS AND METHODS

### 2.1 Plasmid construction and Agrobacterium transformation

The superbinary plasmid pEPS3004 was constructed for transformation of maize through recombination of pSB1 binary plasmid and pSB11 plasmid derivative—pEPP3028. The pEPP3028 was introduced into *Agrobacterium tumefaciens* strain LBA4404(pSB1) (Komari et al., 2006; Komori et al., 2007) and the structure of the pSB1::pEPP3028 co‐integrate superbinary plasmid, pEPS3004, was validated by restriction enzyme digestion and DNA sequence analysis of selected segments. The T‐DNA in the transformation vector, pEPS3004, contains the elements of the activation tagging sequences; (a) the non‐autonomous transposon comprised of the 4X *SCBV* enhancer, the *OsAct1p*::AAD1 selectable marker and the terminal inverted repeats (TIR) from the *Spm* transposon, (b) the transcription factors controlling anthocyanin biosynthesis, *ZmGLOB1*::*B‐peru* and *ZmGLOB1*::*C1*, and (c) the *Spm* transposase. The 4X *SCBV* enhancer and the *OsAct1p*::AAD1 selectable marker were subcloned from pDAB3878 as described in Davies et al. (2014). The *ZmGLOB1*::*B‐peru* and *ZmGLOB1*::*C1* genes were PCR amplified from the vector pDAB2125 as described in Petolino and Shen (2006). The *Spm* TIRs and transposase were synthesized at DNA2.0 (now ATUM) according to the description in Kumar et al., 2005 using sequences from GenBank Accession M25427.1.

### 2.2 Particle bombardment of maize embryos

Maize ears were collected 13 days after pollination (DAP) and surface sterilized with 50% bleach for 15 min, followed by five washes with sterile distilled water. Embryos were isolated using sterile forceps and 30 embryos were placed on a Petri plate containing MS medium (Murashige & Skoog, 1962) with 3% sucrose for 24 hr in darkness at 28°C. On the day of bombardment, the embryos were moved to MS medium containing 12% sucrose and incubated in darkness at 28°C for 4 hr prior to bombardment.

Gold particles (1 μm diameter) were washed with 70% ethanol for 10 min, then rinsed three times with sterile water. The particles were dispensed in 50% glycerol at a concentration of 120 mg/ml. To 50 μl (6 mg) of gold particles, 5 μg of plasmid DNA, 50 μl of 2.5 M CaCl_2_ and 20 μl 0.1 M spermidine were added. The reaction (total volume 125 μl) was incubated at room temperature for 10 min with gentle shaking, then for another 10 min without shaking. The DNA coated‐gold particles were briefly centrifuged, washed with 150 μl of 70% ethanol and then with 100% ethanol. The final pellet was resuspended in 30 μl of 100% ethanol and subjected to a brief sonication with a Branson 3510 sonicator. A 10 μl aliquot of the gold‐particles coated with DNA was spread on macrocarriers (BioRad, Hercules, CA) and used in bombardment assays using a BioRad PDS1000/He system. The embryos were transformed at a target distance of 6 cm using 1,100 psi disks. Following bombardment, the embryos were moved to MS medium containing 3% sucrose and incubated under light (approximately 50 μmoles m^−2^s^−1^) for 48 hr at 28°C. Accumulation of anthocyanin pigments was observed under a light microscope.

### 2.3 Maize transformation

Seeds from the B104 inbred were planted into 4‐gallon‐pots containing Sunshine Custom Blend^®^ 160 (Sun Gro Horticulture, Bellevue, WA). The plants were grown in a greenhouse using a combination of high pressure sodium and metal halide lamps with a 16:8 hr Light:Dark photoperiod. To obtain immature embryos for transformation, controlled sib‐pollinations were performed. Immature embryos were isolated at 10 to 13 days after pollination when embryos were approximately 1.4 to 2.0 mm in size.

Prior to embryo excision and transformation, maize ears were surface sterilized by immersing them in 50% commercial bleach with Tween 20 (1 or 2 drops per 500 ml) for 10 min and rinsed three times with sterile water. A suspension of *Agrobacterium* cells containing a superbinary vector was prepared by transferring one or two loops of bacteria grown on YEP (5 g/L yeast extract, 10 g/L peptone, 5 g/L sodium chloride, 15 g/L Bacto Agar) solid medium containing 50 mg/L spectinomycin, 10 mg/L rifampicin, and 50 mg/L streptomycin at 28°C for 3 days or 25° for 4 days into 5 ml of liquid infection medium (MS salts, ISU Modified MS Vitamin stock (1,000×, 2 g/L glycine, 0.5 g/L each of thiamine HCl and pyridoxine HCl, 0.05 g/L nicotinic acid (Frame et al., 2006), 3.3 mg/L dicamba, 68.4 gm/L sucrose, 36 gm/L glucose, 700 mg/L l‐proline, pH 5.2) containing 100 μM acetosyringone. The solution was gently pipetted up and down using a sterile 5 ml pipette until an uniform suspension was achieved, and the concentration was adjusted to an optical density of 0.3 to 0.5 at 600 nm (OD_600_) using an Ultrospec 10 Cell Density Meter (GE Healthcare/Amersham Biosciences, Piscataway, NJ). Immature embryos were isolated directly into a micro centrifuge tube containing 2 ml of the infection medium. The medium was removed and replaced twice with 1 to 2 ml of fresh infection medium, then removed and replaced with 1.5 ml of the *Agrobacterium* solution. The *Agrobacterium* and embryo solution was incubated for 5 min at room temperature and then transferred to co‐cultivation medium, which contained (MS salts, ISU Modified MS Vitamin stock (1,000×, 2 g/L glycine, 0.5 g/L each of thiamine HCl and pyridoxine HCl, 0.05 g/L nicotinic acid, 3.3 mg/L Dicamba, 68.4 gm/L sucrose, 36 gm/L glucose, 700 mg/L l‐proline, pH 5.2) containing 100 μM acetosyringone. Co‐cultivation incubation was for 3 to 4 days at 25° under either dark or 24‐hr white fluorescent light conditions (approximately 50 μmoles m^−2^s^−1^).

After co‐cultivation, the embryos were transferred to a non‐selection MS salts, ISU Modified MS Vitamins, 3.3 mg/L Dicamba, 30 gm/L sucrose, 700 mg/L l‐proline, 100 mg/L myo‐inositol, 100 mg/L Casein Enzymatic Hydrolysate, 15 mg/L AgNO_3_, 0.5 gm/L MES (2‐(N‐morpholino)ethanesulfonic acid monohydrate; Fischer Scientific, Waltham, MA), 250 mg/L Carbenicillin, and 2.3 gm/L Gelzan™, at pH 5.8. Incubation was continued for 7 days at 28°C under either dark or 24‐hr white fluorescent light conditions (approximately 50 μmoles m^−2^s^−1^). Following the 7‐day resting period, the embryos were transferred to selective medium (the MS‐based resting medium (above) supplemented with haloxyfop). The embryos were first transferred to selection medium containing 100 nM haloxyfop and incubated for 1 to 2 weeks, and then transferred to selection medium containing 500 nM haloxyfop and incubated for an additional 2 to 4 weeks. Transformed isolates were obtained over the course of approximately 5 to 8 weeks at 28°C under either dark or 24‐hr white fluorescent light conditions (approximately 50 μmoles m^−2^s^−1^).

Following the selection process, cultures were transferred to an MS‐based pre‐regeneration medium containing MS salts, ISU Modified MS Vitamins, 45 gm/L sucrose, 350 mg/L l‐proline, 100 mg/L myo‐inositol, 50 mg/L Casein Enzymatic Hydrolysate, 1 mg/L AgNO_3_, 0.25 gm/L MES, 0.5 mg/L naphthaleneacetic acid, 2.5 mg/L abscisic acid, 1 mg/L 6‐benzylaminopurine, 250 mg/L Carbenicillin, 2.5 gm/L Gelzan™, and 500 nM Haloxyfop, at pH 5.8. Incubation was continued for 7 days at 28°C under 24‐hr white fluorescent light conditions (approximately 50 μmoles m^−2^s^−1^).

For regeneration, the cultures were transferred to an MS‐based primary regeneration medium containing MS salts, ISU Modified MS Vitamins, 60 gm/L sucrose, 100 mg/L myo‐inositol, 125 mg/L carbenicillin, 2.5 gm/L Gelzan™, and 500 nM haloxyfop, at pH 5.8. After 2 weeks at 28°C under either dark or 24‐hr white fluorescent light conditions (approximately 50 μmoles m^−2^s^−1^), tissues were transferred to an MS‐based secondary regeneration medium composed of MS salts, ISU Modified MS Vitamins, 30 gm/L sucrose, 100 mg/L myo‐inositol, 3 gm/L Gelzan™, at pH 5.8, with, or without, 500 nM haloxyfop. Regeneration/selection was continued for 2 weeks at 28°C under either 16‐hr or 24‐hr white fluorescent light conditions (approximately 50 μmoles m^−2^s^−1^). When plantlets reached 3 to 5 cm in length, they were excised and transferred to secondary regeneration medium (as above, but without haloxyfop) and incubated at 25°C under 16‐hr white fluorescent light conditions (approximately 50 μmoles m^−2^s^−1^) to allow for further growth and development of the shoot and roots.

Plants were transplanted into Metro‐Mix^®^ 360 soil‐less growing medium (Sun Gro Horticulture) and incubated in a growth room. Plants were then transplanted into Sunshine Custom Blend 160 soil mixture and grown to flowering in the greenhouse. Controlled pollinations for seed production were conducted.

### 2.4 Genomic DNA isolation and PCR amplification of excision sites

Genomic DNA was isolated using Qiagen DNAeasy kits. Polymerase Chain Reaction (PCR) (Mullis et al., 1986) was performed using the forward primer (5′‐GTACCTCTTCCTGGAGCACCAG‐3′) which hybridized to sequences between the 13,961 bp and 13,982 bp pEPS3004 and the reverse primer (5′‐TGTAGAACCCGTCCGTCCGTCCACGTCAG‐3′) which hybridizes to sequences between 20,359 bp and 20,383 bp in pEPS3004. PCR products were cloned into a TOPO vector (Invitrogen, Carlsbad, CA) and transformed into *Escherichia coli* cells. Plasmid DNA was prepared from isolated colonies and sequenced by Sanger dideoxy sequencing (Sanger, Nicklen, & Coulson, 1977).

### 2.5 Field growth of pEPS3004 transformed plants

In 2010, T1 seed from 68 single copy events containing the T‐DNA from pEPS3004 were planted at the DAS field station in Molokai, HI. Prior to planting, the field was prepared by irrigation and application of glyphosate. All field operations followed the DAS Regulated Transgene Standard Operating Procedures (RTSOP). Ten‐foot rows were planted by alternating four rows of transgenic plants and four rows of non‐transgenic B104. All transgenic event rows were sprayed with Quizalofop (Assure^®^II) at 2–3 leaf stage to confirm selectable marker segregation and eliminate herbicide susceptible plants. Fertilizer was applied once every 2 weeks through irrigation water to provide 300–400 pounds of nitrogen per acre throughout the lifetime of a plant. Transgenic plants were detasseled prior to pollen shed to assure pollination was from non‐transgenic B104. Ears were harvested from individual plants and kept separate by ear. Kernels were classified as yellow, yellow with purple sectors or purple and number of kernels with each phenotype were tabulated by ear.

In 2011 T2 seed from 21 of the 68 events grown in 2010 was planted at the Gray Research Production field site in Ashkum IL. The field was prepared applying the herbicides Impact^®^ and Atrazine and fertilized with 150 lbs/acre potash 150 lbs/acre diammonium phosphate and 150 lbs/acre nitrogen. After herbicide and fertilizer application, the field was plowed. All field procedures were performed following DAS Regulated Transgene Standard Operating Procedures (RTSOP). Fifteen‐foot rows were planted alternating four rows of transgenic plants and two rows of non‐transgenic B104. All transgenic plants were sprayed with Quizalofop (Assure^®^II) at the 2–3 leaf stage and detasseled prior to pollen release. Mature ears were harvested, shelled, and sent to DAS.

### 2.6 Next‐Generation Sequencing (NGS) procedures and data analysis

To characterize the site of integration of the T‐DNA from pEPS3004 in the genome (launching sites) as well as the site of re‐insertion of the transposon, the Sequence Capture‐based NGS Event Characterization (EC) pipeline was used following the protocol described in (Guttikonda et al., 2016). Genomic DNA was extracted and sheared to 800 bp fragments, then subjected to hybridization with 50–120 bp overlapping probes that are complementary to construct sequences. The resulting DNA libraries were sequenced with 250 bp paired‐end (PE) reads on an Illumina MiSeq sequencer in 96‐plex pools.

The standard EC computational analysis pipeline was used to characterize the T‐DNA integration site (Guttikonda et al., 2016). The process relies on T‐DNA and genome junction‐spanning paired‐end reads as well as individual reads that span the junctions. By identifying the junction position in multiple fragments, the genomic location of the T‐DNA is determined. Similarly, the re‐insertion site of the transposon is determined by identifying paired‐end read and reads that span the junctions between the transposon and insertion site.

To confirm the T‐DNA and transposon re‐insertion sites identified from the EC computational analysis, a parallel analysis was conducted. We separately used the enhancer‐carrying transposon sequences and the T‐DNA sequences (without the transposon) and ran two analyses separately; the T‐DNA from pEPS3004 without transposon combined with maize genome, and the transposon combined with maize genome. If the intact T‐DNA integrated into the genome and the transposon inserted into a new location, there would be a gap at the site where transposon left the T‐DNA. Both launching site and transposon insertion sites identified in this analysis should be identical with locations from previous combined analyses. This parallel analysis identified gapped reads left by the excision of transposon from T‐DNA and confirmed both the transposon launch site and the transposon re‐insertion site identified in the EC computational analysis.

Finally, the tag sites were compared with current gene annotations for maize genome (Schnable et al., 2009) to investigate the spatial relationships between tag sites and known genes. In particular, the distances between the tag sites and the closet start codons were calculated and summarized (see Section 3).

## 3 RESULTS

### 3.1 Construct used to develop the activation tagging population

**Figure 1:**
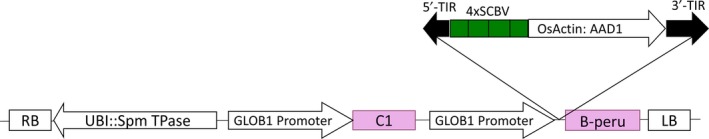
Schematic representation of the T‐DNA in pEPS3004. *Ubi*::*Spm* TPase represents the maize *Ubiquitin 1* promoter driving the *Spm* transposase gene. *GLOB1* Promoter represent the maize *Globulin 1* promoter. *C1* and *B‐peru* represent the coding sequences of the maize anthocyanin transcription factors *C1* and *B‐peru*. 5′TIR and 3′TIR represent the terminal inverted repeats of the *Spm* transposon. 4x*SCBV* represent four copies of the *SCBV* enhancer sequence. *OsActin*:AAD1 represents the rice actin promoter driving expression of the AAD1 herbicide tolerance gene. The 5′TIR: 4x*SCBV*:* OsActin*:AAD1: 3′TIR is the non‐autonomous transposable element located in the 5′ UTR of the *GLOB1* Promoter: *B‐peru* gene

The vector pEPS3004 was used in transformation and transfection experiments described below. This T‐DNA vector is comprised of a non‐autonomous *Spm* transposable element containing the 5′ and 3′ terminal inverted repeats (TIRs) of the *Spm* element (Kumar et al., 2005), four copies of the *SCBV* enhancer sequence (Braithwaite, Geijskes, & Smith, 2004; Davies et al., 2014) and a plant selectable marker composed of the rice actin1 promoter (*OsActin*) (McElroy, Zhang, Cao, & Wu, 1990) and the AAD1 gene, which provides resistance to the herbicide haloxyfop (Wright et al., 2010). To mobilize the non‐autonomous transposable element, the T‐DNA vector also contains the *Spm* transposase (TPase) gene driven by the maize ubiquitin promoter (UBI) (Toki et al., 1992). A schematic of the T‐DNA from this vector is shown in Figure 1 and the sequence of the vector is disclosed in Supporting Information.

In order to observe whether the non‐autonomous transposable element has excised in seed tissues, the engineered transposon was inserted in the 5′UTR of the *ZmGLOB1*::*B‐peru* gene. The *B‐peru* and *C1* genes encode transcription factors that regulate anthocyanin biosynthesis (Bodeau & Walbot, 1992; Grotewold et al., 1998; Ludwig, Bowen, Beach, & Wessler, 1990) and their expression has been shown to be sufficient, in certain genetic backgrounds, for the accumulation of anthocyanins in the embryo and aleurone layer of kernels when driven by the *ZmGLOB1* promoter (Petolino & Shen, 2006). The *B‐peru* and *C1* genes are either absent or not functional in the wild‐type B104 inbred, the maize genotype into which the construct was transformed. Disrupting the *ZmGLOB1*::*B‐peru* gene with the transposable element prevents accumulation of *B‐peru* and anthocyanins. Excision of the non‐autonomous transposable element removes the disruption and repairs the *ZmGLOB1*::*B‐peru* gene resulting in anthocyanin production in tissues where the *ZmGLOB1* promoter functions.

### 3.2 Transposon excision in transient assays

**Figure 2:**
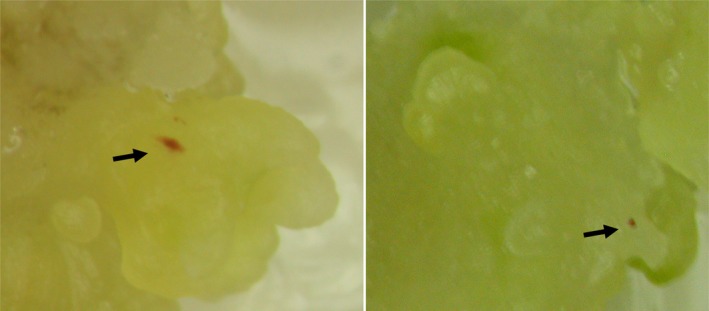
Examples of transient assays showing excision of transposon from pEPS3004. The purple sectors indicate site of excision

To test whether excision of the transposon from the *ZmGLOB1*::*B‐peru* gene causes accumulation of anthocyanin, maize B104 embryos were transfected with pEPS3004 by particle bombardment. The transfected embryos were analyzed for the accumulation of anthocyanin pigments (Figure 2). In two experiments, 3 of 250 and 7 of 250 embryos showed sectors of anthocyanin accumulation suggesting that anthocyanin accumulation resulted in tissues where the transposable element is excised from the disrupted *B‐peru* gene and repaired its structure to a functional *B‐peru* gene.

To determine whether the transposon had excised from the *ZmGLOB1*::*B‐peru* gene in these transient assays, genomic DNA was isolated from the purple sectors, and sequences flanking the transposon in pEPS3004 were amplified by the Polymerase Chain Reaction (PCR). PCR was performed using opposing primers which are approximately 6.4 kbp apart in the intact construct and ~247 bp apart after transposon excision. PCR reactions from samples collected from the purple segments of the callus produced ~247 bp DNA fragments. These PCR products were cloned and sequenced. Six types of fragments were obtained and are summarized in Table 1. These types of mutations have also been observed in other studies characterizing transposon excision sites (Kumar et al., 2005).

**Table 1:** Sequence of 47 empty donor sites (DS) after excision of artificial transposon

pEPS3004 DS	cagtgt[transposon]acgagaca
Empty DS 1 (4)	cagtgt‐‐‐---‐‐‐---acgagaca
Empty DS 2 (39)	cagtgt‐‐‐---‐‐‐---.cgagaca
Empty DS 3 (1)	cagtgt‐‐‐---‐‐‐---.cgagacg
Empty DS 4 (1)	cagtgt‐‐‐---‐‐‐---.tgagaca
Empty DS 5 (1)	cagcgt‐‐-‐‐‐---‐‐‐.cgagaca
Empty DS 6 (1)	cagtg.‐‐---‐‐‐---‐.agaca

The numbers in parentheses indicate number of clones having the indicated sequence in the first and second studies. The dots and underlined bases indicate base pair deletions and transitions, respectively, in the sites. Most of the products identified showed deletions or mutations around the site of excision.

### 3.3 Transposon excision in T1 kernels

The construct pEPS3004 was transformed into maize inbred B104 by Agrobacterium‐mediated immature embryo transformation and transformants were selected by resistance to haloxyfop. Primary T0 transformants were grown to maturity and backcrossed to wild‐type B104 plants to produce T1 seed. A total of 584 herbicide tolerant plants were generated. Transformants with a low copy number, as determined by TaqMan analysis, were subjected to DNA gel blot analysis. Three hundred and twenty‐two of these plants were confirmed to have the AAD1 selectable marker gene and 311 of these plants produced seed (Supporting Information Table S1. T1 Kernel Phenotypes).

**Figure 3:**
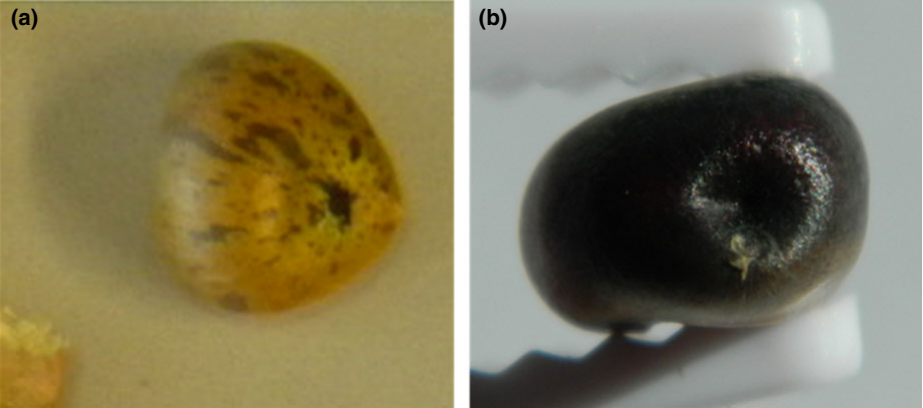
Kernel phenotypes showing (a) purple sectors and (b) purple kernels

Anthocyanin accumulation in the kernels of the T1 ears is an indication that the transposon excised from the *ZmGLOB1:*:*B‐peru* gene. The pattern of anthocyanin accumulation provides an indication of when the excision occurred. Kernels with purple sectors on a yellow background (Figure 3a) are indicative of somatic excision during kernel development, while kernels that are completely purple (Figure 3b) are indicative of an excision event occurring in germinal cells prior to seed development or in somatic cells of developing kernels prior to differentiation of the aleurone layer.

**Figure 4:**
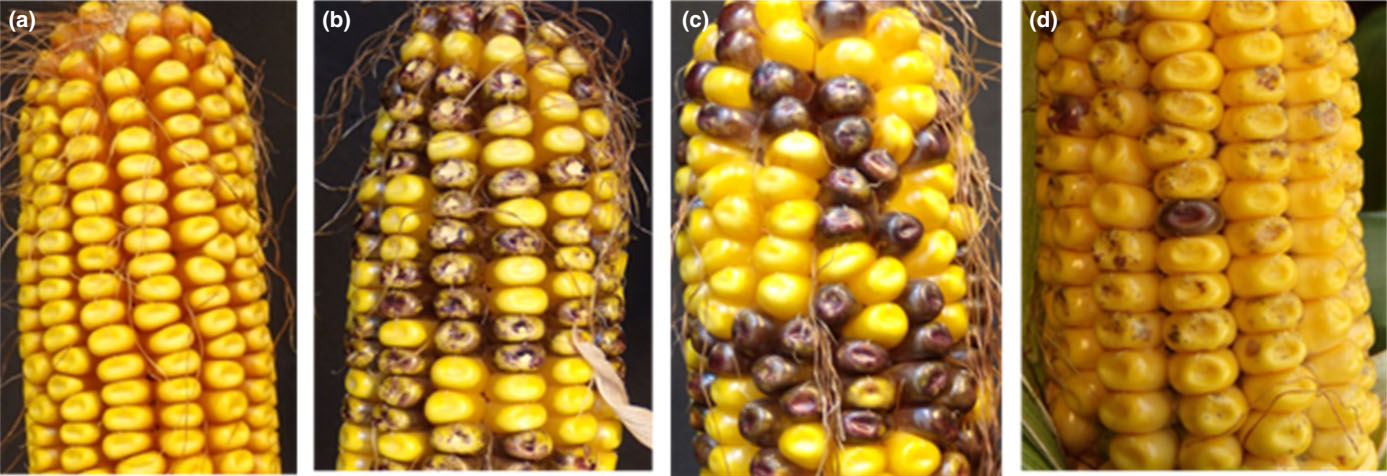
Ear phenotypes from T0 plants. (a) An ear with all yellow kernels. (b) An ear showing ~50% yellow kernels and ~50% yellow kernels with purple sectors. (c) An ear showing ~50% yellow kernels and ~50% solid purple kernels. (d) An ear showing ~50% yellow kernels and ~50% yellow kernels with purple sectors plus a few solid purple kernels

**Figure 5:**
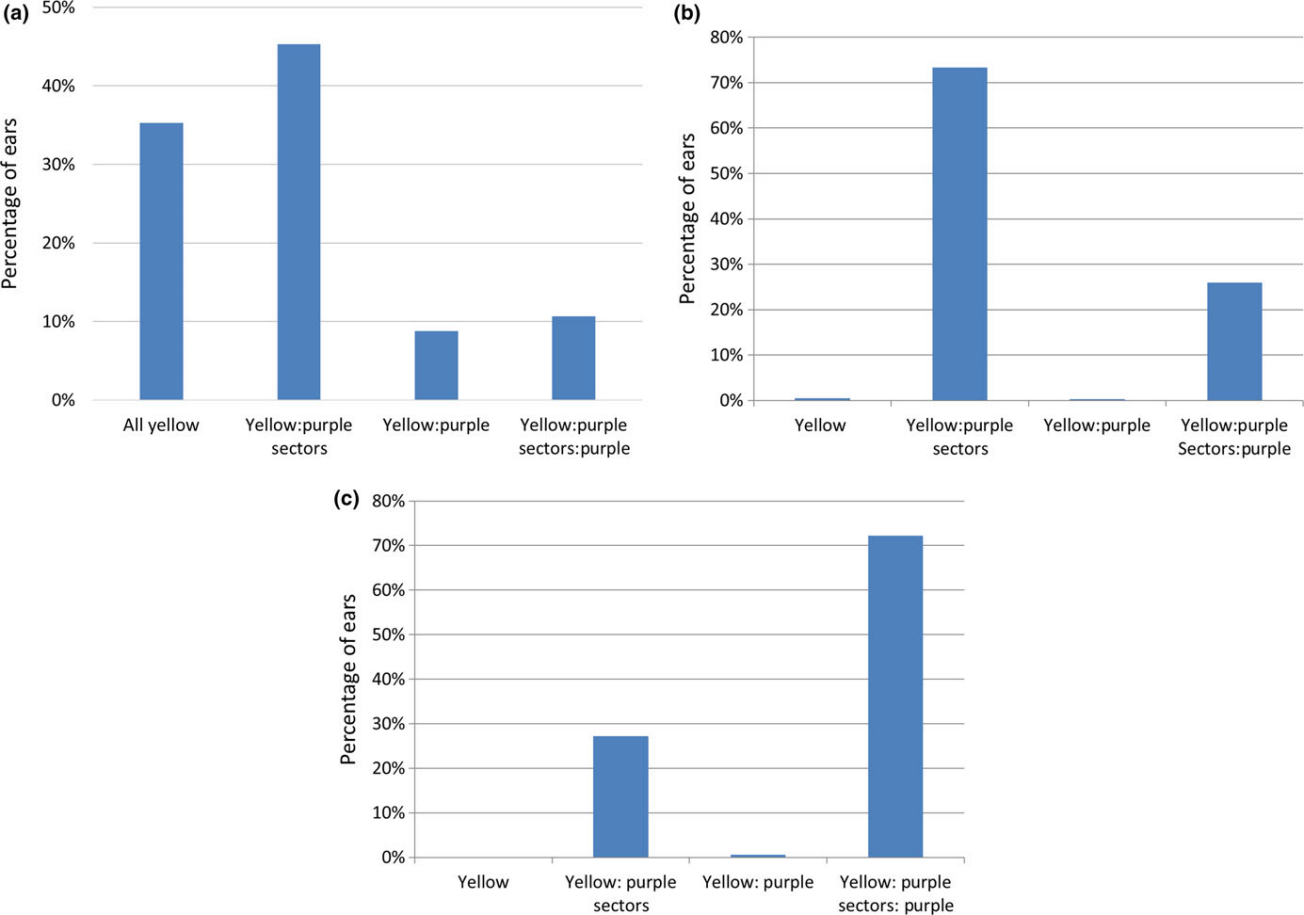
Distribution of ear phenotypes in pEPS3004 events in T1 (a), T2 (b) and T3 (c) generations

The pattern of inheritance of the seed color phenotype in the ear also provides information on transposon excision. Examples of the T1 ear phenotypes are displayed in Figure 4. Because the transgene is in the hemizygous state in the T0 plant and the T0 plants are pollinated with non‐transformed B104, half of the kernels on the ear of a plant should be non‐transgenic and exhibit the wild‐type (yellow) phenotype, while the other half of the kernels should contain the T‐DNA from pEPS3004 in the hemizygous state. Based on when and where excision of the non‐autonomous transposable element occurs, four classes of ear phenotype are observed: (a) when excision of the non‐autonomous transposable element does not occur or when the excision does not result in a functional copy of the *ZmGLOB1*::*B‐peru*, the transgenic kernels will not accumulate anthocyanin and the entire ear will have yellow kernels (Figure 4a), (b) when excision occurs in somatic tissues and results in functional copies of the *ZmGLOB1*::*B‐peru*, the ear should have 50% yellow kernels and 50% yellow kernels with purple sectors (Figure 4b), (c) when excision occurs prior to the germ‐line being established, 50% of the kernels will be yellow and 50% of the kernels purple (Figure 4c), and (d) it is also possible for some of the kernels to be yellow with purple sectors and others to be entirely purple (Figure 4d). Since excision can occur in T0 tissues prior to the development of the T1 ovule or in T1 somatic tissues prior to aleurone tissue differentiation, the purple kernel phenotype may or may not indicate a germinal excision event. Two hundred and twelve of the plants that had been confirmed to be transgenic showed evidence of transposon excision (Supporting Information Table S1. T1 Kernel Phenotypes). Figure 5a shows the distribution of seed phenotypes in the T1 ears of the primary transformants.

**Figure 6:**
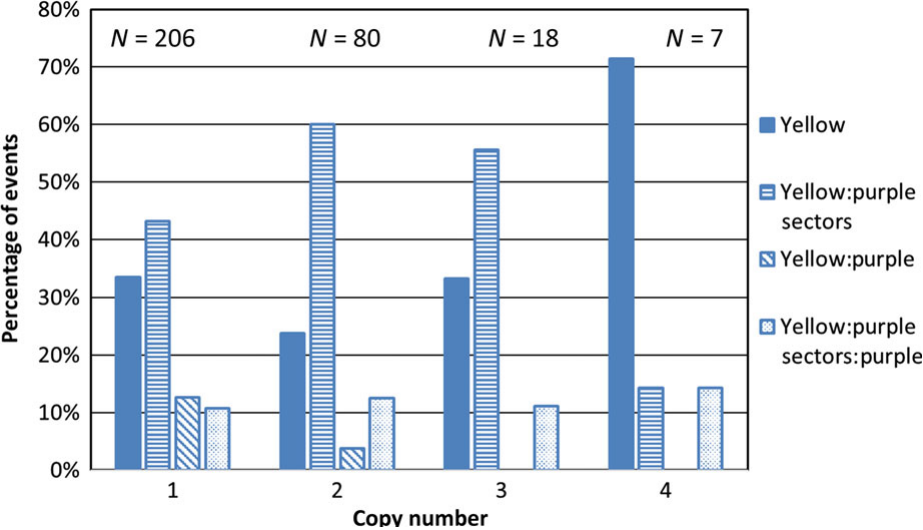
Distribution of T1 ear phenotypes in events with different copy number as determined by DNA gel blot analysis. The number of samples in each category are listed above the bars

To determine whether the rate of excision is affected by the copy number of the T‐DNA inserts, the data are presented as a function of the copy number. The percentage of events in each phenotypic class is shown in Figure 6. Three hundred and eleven events were examined, 206 with one insert, 80 with two inserts, 18 with three inserts, and 7 with four inserts. In events with a single insert, ears with yellow kernels with purple sectors and only yellow kernels are most commonly observed, ears with yellow and purple kernels, and yellow kernels with purple sectors along with a few purple kernels are seen less frequently. The pattern for plants with two or three inserts is similar except that there are fewer ears showing yellow and purple kernels. The patterns suggest that copy number, up to three copies, does not dramatically affect somatic transposon excision but may have some effect on germinal transposon excision rates since the percentage of ears with just yellow and purple kernels declines as the copy number increases. More than three copies may affect transposon excision as five of the seven events with four copies showed no transposon excision but this is based on a very small sample set.

### 3.4 Transposon excision in T1 and T2 generations

To evaluate whether the transposon remained active in the T1 to T2 generation and to increase the amount of seed containing active transposons, approximately 5,000 T1 kernels from 68 randomly selected events showing somatic excisions in the T0 to T1 generation (yellow seed with purple sectors) were planted. Approximately 95% of the seeds germinated and seedlings were sprayed with Assure II (the AAD1 herbicide resistance gene provides resistance to Assure II containing quizalofop) to remove any non‐transgenic seedlings that may have been in the population. As expected, no seedlings were sensitive to Assure II. The plants were grown to maturity, detasseled and backcrossed to B104 to generate T2 seed. Ears were harvested from approximately 4,000 plants from 67 events (one event did not produce any seed). Kernels from these ears were characterized for seed phenotypes as before (Supporting Information Table S2. T2 Kernel Phenotypes). The distribution of phenotypes of ears from T1 plants is shown in Figure 5b.

These results show that 99.7% of the ears had purple sectored or purple kernels indicating events showing active excision in the T0 to T1 generation produced plants that continued to show excision in the T1 to T2 generation; only 10 ears were observed with only yellow kernels indicating no excision occurred or that the excision destroyed the *B‐peru* gene. Furthermore, 26.3% of the ears had one or more purple kernels indicating a putative germinal excision event (18 with ~50% yellow and ~50% purple kernels and 1,017 with yellow, purple sectored and purple kernels).

**Figure 7:**
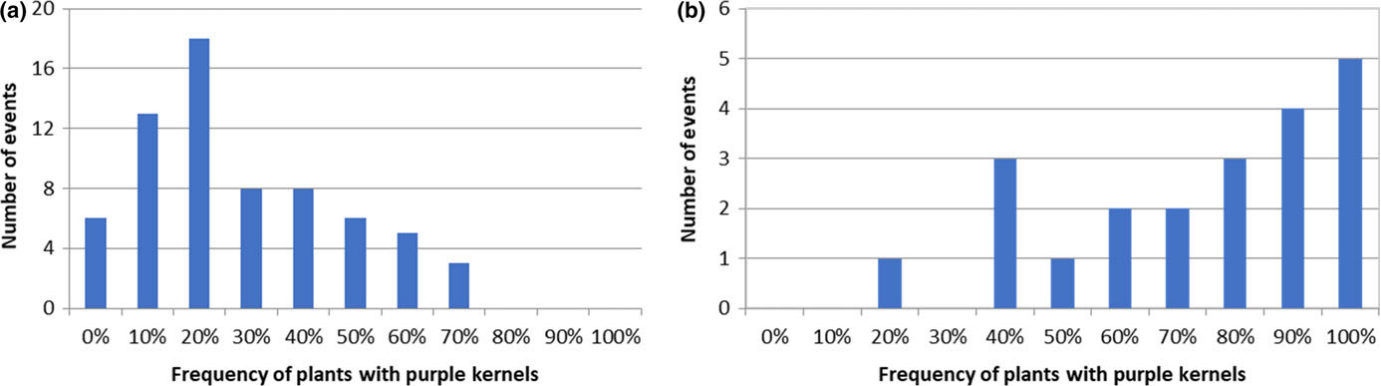
Frequency of putative germinal excision in events in the T1 to T2 (a) and T2 to T3 (b) generations

A goal of this initial analysis was to identify events that show a high rate of germ‐line excision since these types of events are needed for development of the activation tagged population. To efficiently generate the activation tagged population it is desirable to identify events that show relatively high rates of germinal transposition so that fewer parental lines can be used to generate independent transposition events. To identify events that may display a high rate of germinal excision, T2 ears from each event were evaluated for purple kernels. Since purple kernels on the same ear may result from the same excision event, events were evaluated for plants with at least one purple kernel. Figure 7a shows that in some events, very few plants had ears with at least one purple kernel, while in other events, a large percentage of the plants gave rise to ears with at least one purple kernel. In plants grown from the T1 to T2 generation, 44% of the 67 events showed purple kernels arising at a frequency of at least 30% (i.e., at least 30% of the plants from that event produced ears with at least one purple kernel) suggesting that plants from these events show high rates of germinal excision.

### 3.5 Transposon excision in the T2 and T3 generations

To determine whether germinal excision frequencies remained high in the T2 to T3 generation, purple sectored kernels from events exhibiting purple kernel formation in the T1 to T2 generation were grown from the T2 to the T3 generation. Purple sectored kernels from 150 T2 ears from 21 events displaying at least one purple kernel on 30% or more of the ears from the T2 generation were planted in the field and crossed with wild‐type B104. Seed from approximately 10,000 T2 plants were recovered and ~20 randomly selected ears from each event were analyzed for kernel phenotypes (Supporting Information Table S3. T3 Kernel Phenotypes). The results (Figure 5c) show that 27% of the examined ears had yellow kernels, and yellow kernels with purple sectors, in approximately equal numbers. Another 72% of the ears had yellow kernels, yellow kernels with purple sectors, and purple kernels and 1% of the ears had yellow and purple kernels in equal numbers. These results indicate that the transposon remained active in all the ears examined and that 73% of the ears had at least one purple kernel indicating that germinal excision or somatic excision very early in development occurred. These results indicate that excision is stable over at least three generations and that most of the events with progeny showing greater than 30% of the ears with at least one purple kernel in the T1 to T2 maintained a high rate of purple kernel production in the T2 to T3 generation.

To determine whether different events show different rates of purple kernel production, the percentage of ears from each event with at least one purple kernel was determined and plotted in Figure 7b. The results show that most of the events show a high frequency of ears with one or more purple kernel, suggesting that the germinal excision frequency remained high in the T2 to T3 generation. In fact, the frequency of plants observed with at least one purple kernel is greater in the T3 generation than it was in the T1 to T2 generation. The cause of the increase in frequency of purple kernel occurrence is unclear but could be the effect of the environment that is known to influence transposon excision (Hashida, Kitamura, Mikami, & Kishima, 2003). Furthermore, the frequency of ears with purple kernels appeared different across different events (Figure 7b). This suggests that germinal excision is higher in some events than in others and that there may be an influence of the site in the genome from which the transposon launches on the rate of purple kernel formation.

**Figure 8:**
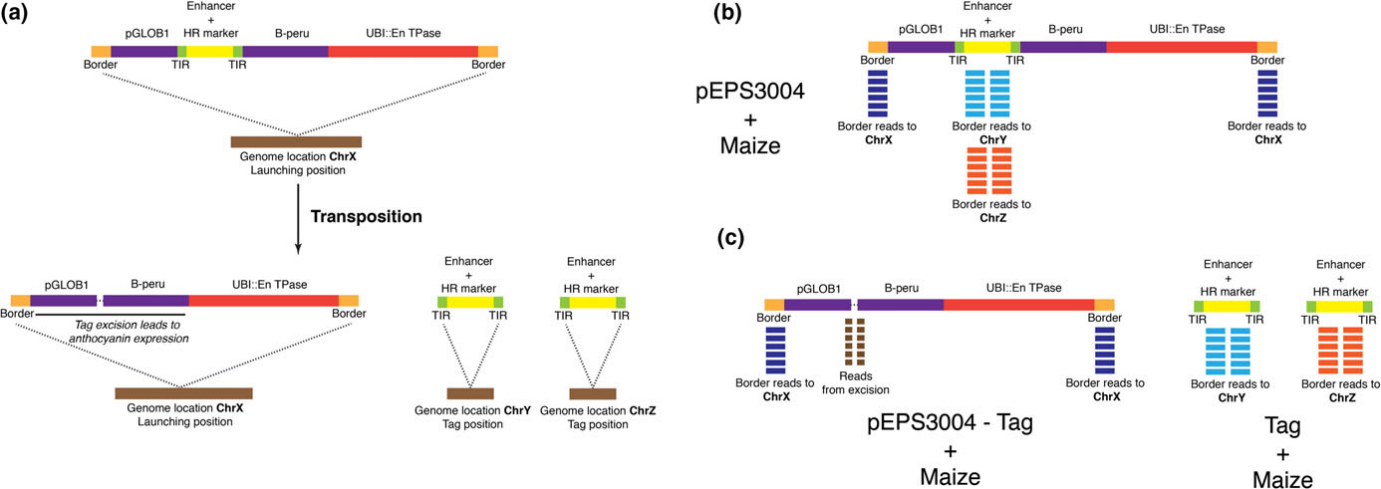
Identification of junction read pairs and individual reads to characterize launching and tag sites. (a) Schematic of sequence analysis of the pEPS3004 T‐DNA prior to and after excision of the transposon. The brown bar represents chromosomal sequences where the T‐DNA integrated (designated on Chromosome X) after excision and re‐integration the transposons integrate on Chr Y and ChrZ. (b) Schematic of sequence analysis of the pEPS3004 insert and the non‐autonomous transposon containing the Enhancer and Herbicide Resistance (HR) marker after transposon excision. (c) The genomic location of the T‐DNA is determined (blue reads which map to chrX. The genomic location of the site of transposon re‐insertion is determined by identifying reads that span the junctions between the transposon and insertion site (light blue and orange reads to chrY, chrZ)

### 3.6 Phenotypic analysis of plants derived from purple kernels

Purple kernels can arise either from germinal excision events or from somatic excision events that occur prior to the development of the aleurone layer. Aleurone tissue is derived from endosperm and the embryo and endosperm have separate cell lineages; the embryo is derived from the fusion of a sperm cell nucleus and the central nucleus of the central cell whereas endosperm along with aleurone is formed from the fusion of a sperm cell nucleus and two polar nuclei in the central cell (Becraft & Yi, 2010). This means that a purple aleurone can be derived from a germinal excision event prior to the formation of the female gametophyte or sometime after the fusion of the sperm cell nucleus and the two polar nuclei that give rise to the endosperm but before differentiation of the aleurone layer. Furthermore, since aleurone and embryo cell lineages separate very early in kernel development, it is possible for an embryo in a purple kernel to contain a transposon in the launch site. The phenotypes of kernels in the subsequent generation can distinguish between germinal and early somatic excision events. Plants derived from purple kernels that produce 50% yellow and 50% purple seed are consistent with germinal excision (and not early somatic excision) because it is highly improbable that half the kernels on an ear would show somatic excision simultaneously early in embryo development. While plants derived from purple kernels that produce ears with 50% yellow kernels and 50% yellow kernels with purple sectors are derived from somatic transposon excision events.

To determine the frequency of purple kernels representing germinal and somatic excision events, 20 herbicide‐resistant T2 plants derived from purple kernels of ears exhibiting yellow, yellow with purple sectors and purple kernels were grown to maturity and pollinated by non‐transgenic B104 to produce seed (one plant died prior to pollination). Table 2 shows that 12 of the 19 plants produced ears with only yellow kernels and purple kernels in an approximate ratio of 1:1; consistent with the parental seed being derived from a germinal excision event. The other seven plants produced ears with yellow and purple sectored kernels in roughly equal numbers with a few purple kernels; consistent with the parental seed being derived from a somatic excision event. In this experiment ~60% of the kernels classified as purple (on ears with yellow, yellow with purple sectors and purple kernels) result from germinal excision events. The results also indicate that purple kernels on the same ear may be derived from either a germinal or a somatic excision event. These results need to be considered when identifying kernels representing germinal excision events.

**Table 2:** Analysis of kernel phenotypes on plants derived from purple kernels on ears containing yellow, purple sectored and purple kernels

Plant ID	Event	Total kernels	Yellow	Purple sectored	Purple	Transposon excision in previous generation
ZT00326958	ZX7597‐0335382	453	241	0	212	Germinal
ZT00326959	ZX7597‐0335382	435	221	0	214	Germinal
ZT00326964	ZX7597‐0335382	430	209	0	221	Germinal
ZT00326965	ZX7597‐0335382	498	251	0	247	Germinal
ZT00326966	ZX7597‐0335382	514	254	0	260	Germinal
ZT00326968	ZX5895‐0210552	426	218	205	3	Somatic
ZT00326970	ZX5895‐0210552	174	83	0	91	Germinal
ZT00326971	ZX5895‐0210552	564	276	0	288	Germinal
ZT00326974	ZX5895‐0210552	502	246	248	8	Somatic
ZT00326976	ZX8617‐0405571	216	94	121	1	Somatic
ZT00326982	ZX8617‐0405571	326	158	168	0	Somatic
ZT00326984	ZX7678‐0343833	401	198	0	203	Germinal
ZT00326985	ZX7678‐0343833	522	261	0	261	Germinal
ZT00326987	ZX7678‐0343833	463	216	0	247	Germinal
ZT00326988	ZX7678‐0343833	381	177	199	5	Somatic
ZT00326989	ZX7678‐0343833	185	99	0	86	Germinal
ZT00326990	ZX7678‐0343833	451	211	0	240	Germinal
ZT00326991	ZX7678‐0343833	513	255	256	2	Somatic
ZT00326997	ZX4756‐0135876	368	184	183	1	Somatic

The colors are just to distinguish the events. Multiple plants from 4 of the 5 events were examined.

### 3.7 Molecular analysis of transposition

Molecular characterization of transposition was performed on a set of plants derived from purple kernels to identify the launch site of the transposon, whether a plant was derived from germinal or somatic transposition and where the transposon integrated after re‐insertion. To characterize transposon activity in the events generated in this study, 516 purple kernels were collected from 151 T3 ears that had kernels that were yellow, yellow with purple sectors and completely purple. These 151 ears were from 10 events that had shown high rates of ears with purple kernels. The purple kernels were planted, allowed to germinate and DNA was isolated from seedling leaf tissue. PCR analysis was performed to determine which plants contained the AAD1 gene, indicating that they contained the transposon. Three hundred and forty‐nine of the 516 plants examined contained the AAD1 gene. DNA capture and sequencing (Figure 8) were performed to identify the launch site of the transposon (the location of the *B‐peru* gene) and the re‐insertion site of the transposon on 192 of the 349 AAD1 positive plants; these plants were from nine different events. Insertion sites were mapped by identifying sequences adjacent to the inserted sequences and mapping those to genomic sequences of the reference genome.

Launch sites were able to be mapped in six of the nine events (Supporting Information Table S4. Tpn launch integration site), in the other three events the sequences of the transferred DNA were detected but their location in the genome was not able to be determined because sequences adjacent to the inserted DNA contained only repetitive DNA. The launch sites in the six events that were mapped were found on five different chromosomes.

To determine which of the purple kernels were likely the result of a germ‐line or early somatic transposon excision event, the sequences at the launch site were examined to determine whether they still contained the transposon or whether the transposon was absent. Since the transposon can move at any time during the development the launch sites in the sampled leaf tissue may be completely homogenous (containing only launch sites with the transposon either present or absent) or heterogeneous (some cells containing launch sites with the transposon and some without). Tissues containing homogenous launch sites without the transposon may result from a germ‐line excision event or a somatic excision event that occurred prior to the development of the sampled tissue. Tissues containing homogenous launch sites with the transposon present indicate that the anthocyanin accumulation in the kernel was due to somatic excision of the transposon prior to development of the aleurone layer. Tissues that contain heterogeneous launch sites also indicate that the anthocyanin accumulation in the kernel that gave rise to the sampled plant was due to somatic excision prior to development of the aleurone layer. Table 3 shows the results for the sequencing of samples. Of these the 192 plants examined, 38 had all sequencing reads showing empty launch sites, consistent with germ‐line excision of the transposon, 149 had mixed (some reads with the transposon present and some reads absent), and five had all sequencing reads with the transposon present (Table 3). Events varied from 9% to 30% in the frequency of homogenous empty launch sites.

**Table 3:** Launch sites in 192 plants from 9 events containing the activation tagging element

Event	Empty	Mixed	Full	Percent germinal
302	4	19	1	17
213	3	27		10
933	2	16		11
240	4	10		29
439	1	10		9
461	1	3		25
709	3	12		20
800	18	39	3	30
101	2	13	1	13
Total	38	149	5	

Launch sites were examined by DNA capture sequence analysis and the presence or absence of the transposable element determined. Empty indicates that no reads with a transposable element were identified, Mixed indicates that reads with and without the transposable element were identified, and Full indicates that only reads with the transposable element were identified.

**Figure 9:**
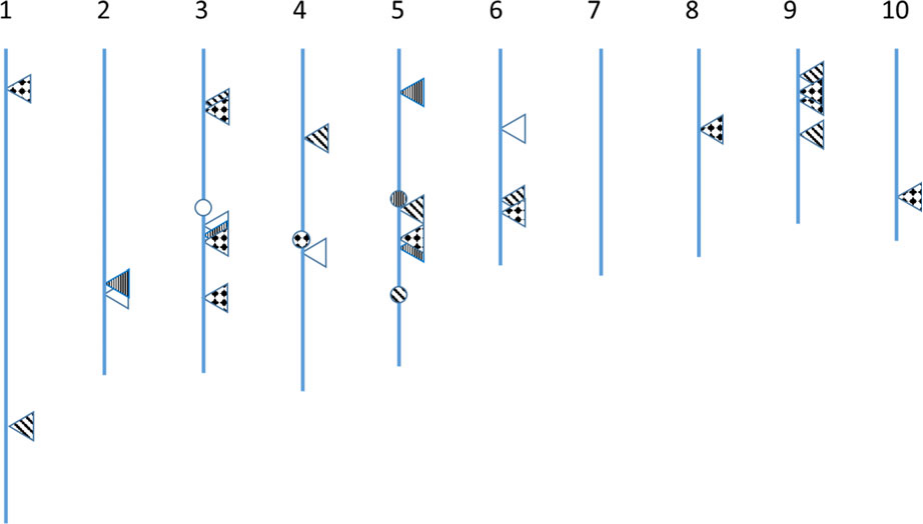
Insertion of the *Spm*‐derived Activation Tagging transposon occurs independent of the launch site. Launch sites are designated by circles and insertion sites are designated by triangles. The patters designate transposons from different launch sites

Since activation tagging has been characterized to be effective on genes near the integration site of the enhancers, it is useful to understand where the transposon moves and how often it integrates near genes. The location of the site of integration of the transposon was mapped in 103 of the 192 samples tested (Supporting Information Table S4. Tpn launch integration site); the definitive site of many of the transposon insertions was hard to place because either no paired‐end sequencing reads were derived that connected sequences of the transposon and the sequences flanking the insertion site, or that the sequences that flank the insertion site contained only repetitive DNA. The frequency of placement of re‐inserted transposons across events is reported in Supporting Information Table S5. Tpn Re‐insert Place Freq. Figure 9 shows transposon re‐insertion sites from four events; the data indicate that the location of transposon insertion appears to be independent of launch site.

The location of the transposon relative to the nearest adjacent gene was also determined. It was found that 20 of the 103 transposons mapped (19%) integrated within the coding sequences of a gene and that 56 of 103 (54%) integrated within 10 kbp of a gene, and 27 of 103 (26%) integrated further than 10 kbp away from the closest gene (Supporting Information Table S6. Re‐insertion site context).

## 4 DISCUSSION

This maize activation tagging platform uses the *En*/*Spm* transposon to distribute the activation tagging element throughout the maize genome. When excision and re‐insertion of the transposon occurs in somatic cells, just those cells and their mitotic products will be tagged but the activation tagging event will not be passed on to the next generation. However, if excision and re‐insertion occurs in a germ‐line cell, the activation tagging event can be passed on to the next generation. Germ‐line excision events do not occur at a high frequency, but the pattern of expression of the excision‐dependent color marker can help to identify germ‐line excision events. When excision occurs in the germ‐line, the anthocyanin inducing *B‐peru* and *C1* genes along with the transposase will be inherited in the next generation. When a line in which a germ‐line excision event has occurred is backcrossed with the non‐transgenic progenitor line, the purple kernel phenotype will be observed in progeny that inherit the anthocyanin and transposase genes. After excision, the transposon can re‐insert in the genome at another location. Because germ‐line cells will undergo meiosis resulting in crossing over, reduction from 2n to 1n chromosome number and independent assortment of chromosomes, the activation tagging element, may not be inherited with the anthocyanin and transposase genes. However, the activation tagging element should be present in approximately 50% of the purple kernels.

Not all excision events that give rise to purple kernels are due to germ‐line excision events. Purple kernels can also be derived from transposon excision in endosperm cells after double fertilization but prior to aleurone layer development. In this case, transposon excision occurs independent of transposon excision in the embryo and will not be inherited in the next generation. Plants derived from these purple kernels will have active transposons that can give rise to either germinal or somatic transposon excision events.

The *En*/*Spm* transposon used in this study has shown activity over three generations. In the first generation 212 of the 311 (68%) primary transformants showed evidence of transposon excision as detected by purple sectors in the aleurone layer of the kernel. Kumar et al. (2005) observed that 65% of primary transformants carrying the *Spm* transposon showed active transposon excision. Sixty‐eight events showing active transposon excision in the T0 to T1 generation were grown in the T1 to T2 generation and showed active transposon excision in that generation as well. Only 18 plants of the 3,949 plants grown in this generation showed no sign of transposon excision. These 18 plants were from 12 separate events. The 20 events that were grown in the T2 to T3 generation also showed active transposon excision; all 515 plants examined from this generation showed evidence of transposon excision.

To identify and characterize transposition events in purple kernels, two separate experiments were performed. In both experiments the purple kernels came from ears that had yellow, yellow with purple sectors and purple kernels. In the first experiment, T2 plants derived from the purple kernels were planted and tested for the presence of the activation tag by PCR analysis. Those that had the activation tagging element, were backcrossed with non‐transgenic B104 and the kernel phenotypes were examined. This experiment showed that 63% (12 of 19 plants) produced ears with 50% purple kernels and 50% yellow kernels indicating a germinal transposon excision event had occurred while the other 37% produced ears with ~50% yellow kernels with purple sectors and 50% yellow kernels (some of these ears had a few purple kernels also), indicating that the purple kernel that gave rise to these lines were not the result of a germinal excision event. In the second experiment, T3 plants derived from the purple kernels were sampled for DNA capture and sequence analysis to determine the sites of both the T‐DNA and the transposon containing the activation tagging element. Plants that result from a germinal transposon excision event should have no activation tagging sequences at the original T‐DNA integration site and ~20% (38 of 192) showed this result. Seventy‐eight percent (149 of 192) showed some sequences at the original integration site and some at other sites and ~3% (6 of 192) showed activation tagging sequences only at the original site of insertion. These data indicate that the purple seed phenotype in 80% of the T3 plants tested in this experiment were derived from somatic transposon excision. Thus, the two experiments gave different results with phenotypic analysis (in the first experiment) indicating that ~60% of the purple seed derived plants resulting from germ‐line excision and the molecular analysis in the second experiment indicating only ~20% of the lines were the result of germinal excision events. The reason for this difference is not apparent but the plants used in the different experiments were derived from different generations and under different environmental conditions. In the second experiment, only slight differences were observed between events with germinal excision frequencies varying between 9% and 30% (Table 3). The *En*/*Spm* transposon has been used to generate mutant collections through germ‐line transposition in rice and Arabidopsis. Kumar et al. (2005) saw that germinal transposon excision frequency in rice varied by event from 0% to 85%, while Marsch‐Martinez et al. (2002) did not report the frequency of germ‐line transposon excision or insertion.

Mapping the location of the T‐DNA and the site of re‐insertion of the transposon was attempted in 192 plant samples that contain the transposon from nine events. T‐DNA sequences were successfully placed for six of the nine events and were placed on five separate chromosomes. The site of re‐insertion of the transposon was determined for 103 lines (54% of those examined). The lack of a placement for 46% of samples may result from only repetitive sequences being detected in the reads from genomic DNA adjacent to the insertion site of the activation tagging element. The structure of the transposon and the method used determine the site of transposon integration may have resulted in lower than expected placement rate for transposon re‐insertions. The transposon is flanked by terminal inverted repeats (TIRs) that are comprised of repetitive sequences that are found throughout the B104 genome. The size of these TIRs is 270 bp and 641 bp. The sequence‐capture process captures DNA fragments with an average size of 800 bp and the paired‐end sequencing process elucidates ~125 bp at the end of these fragments. Because of this, the method has limited ability to resolve integration sites since the fragments must have one end between the TIRs and the other in a non‐repetitive region of the genome. This will occur infrequently when the TIR between these reads is 614 bp of repetitive sequences. It will occur more frequently when the TIR is 270 bp but placement of the sequence is still limited by the 125 bp sequencing read and the genome, which is comprised of about 85% transposable elements (Schnable et al., 2009).

The 103 lines showing placement of the re‐inserted transposed activation tagging element showed that the transposon usually re‐inserted on a different chromosome than where it originated and insertions in all the chromosomes were observed. These placements were mapped relative to the nearest gene and showed that 19% of them inserted within a gene, 54% within 10 kbp of the nearest gene, and 26% greater than 10 kbp of the nearest gene. In rice, 70% of the placements of the *Spm* transposon were within 600 bp of a gene and 70% of those were within the coding sequences of the gene (Kumar et al., 2005).

A preliminary experiment to determine the rate of plants with aberrant phenotypes was performed by planting purple kernels from ears showing both purple kernels and yellow kernels with purple sectors (i.e., plants most likely to have unique transposon excision events). In this experiment, ~15,000 purple kernels from the T3 generation were planted in the field and 158 plants with aberrant phenotypes were observed but subsequent genetic experiments to link plants with aberrant phenotypes to the activation tagging element have not been performed. Based on experiments noted above, a significant portion of the purple seed may not have resulted from germ‐line excisions and 50% of those with germ‐line insertions are likely to not have inherited the activation tagging element. Nonetheless, the ~1% rate of mutant phenotypes observed is comparable to what was reported in Weigel et al. (2000) and Marsch‐Martinez et al. (2002) but is somewhat less than the 6.4% that Wan et al. (2009) reported.

Future development of the activation tagged population will require the identification of large numbers of germ‐line transposition events by screening for purple kernels on ears that also contain yellow kernels with purple sectors. Each of these purple kernels may represent an independent germinal transposon excision and possible re‐insertion event. The purple kernels from ears with 50% yellow and 50% purple kernels are most likely due to the same germ‐line excision event so the purple kernels from ears that have both purple kernels and yellow kernels with purple sectors will be collected. The T3 plants derived from these purple kernels will be backcrossed to B104 and examined for ear phenotypes. Those that produce a T4 generation with 50% purple kernels and 50% yellow kernels will be kept as germ‐line transposition events. As noted above, the transposed activation tagging element is likely not to be genetically linked with the anthocyanin and transposase gene and will segregate independently. In this case, half of the purple kernels and half the yellow kernels from the T4 generation will have the activation tagging element and half will not. When the transposase and the activation tagging element are in the same cell, the transposon may continue to move. To develop lines where the activation tagging element is stable, lines in which the activation tagging element has segregated away from the transposase need to be identified. These lines can be identified as yellow T4 kernels that are herbicide tolerant; the *OsActin*::AAD1 herbicide tolerance gene is on the activation tagging element. Once these lines have been developed they can be screened for aberrant phenotypes that may be caused by the activation tagging element.

As the earth's population continues to grow, increased food production is needed. Because arable land is not increasing, there is a need to increase the amount of food produced on land currently farmed. Along with enhanced breeding techniques and improved agronomic practices, the discovery of genes that can enhance crop performance is needed. Since no other mutagen efficiently causes the activation of gene expression, the development of an activation tagged population of maize is an unique platform to identify gene function and may lead to the identification of beneficial phenotypes such as tolerance to biotic and abiotic stresses, enhanced nitrogen use efficiency and perhaps increased yield. Creating this activation tagged population in an important crop like maize will avert the problems of translating discoveries made in a model or non‐target crops species into maize and may lead to new maize hybrids with enhanced yield.

## AUTHOR'S CONTRIBUTIONS

DRW, ASR, and JPD conceived of and supervised the work. VR, XLL, WMA, OF, PM, KJ, and JPD designed experiments and analyzed results. VR, XLL, OF, PM, and KJ conducted and supervised the experiments. JPD wrote the manuscript and all the authors read, corrected, and approved the final manuscript.

## Data Availability

Sequences from the molecular analysis of T3 plants have been deposited at NCBI SRA under file names SRR8182367–SRR8182558. Because of material proprietary to Corteva Agriscience, the agricultural division of DowDuPont, the constructs and maize lines containing active transposons are not able to be distributed.
