# Supplementary material for: Development of an activation tagging system for maize
Source: Plant Direct. 2019 Feb 21;3(2):e00118. doi: 10.1002/pld3.118 (PMC6508757; doi:10.1002/pld3.118)
Supplement: Supplementary file 3 [file PLD3-3-e00118-s003.pdf]

Thank you for resubmitting your manuscript. I think there may be a problem with the attachments not coming through, and apologize if it is on our side. The revised manuscript addresses the comments made by the editors, however Reviewer 1 comments that were provided as an attachment may have been missed. These are copied below. In addition, the supplementary file containing the vector sequence did not seem to be included in the Supplemental Data that was uploaded. Please include this sequence file.

Reviewer #1 Comments:
This revised manuscript has been substantially improved both for clarity and succinctness. There are a few instances requiring minor revisions. The word "transposition" is still present in several places where "excision" should be used (for example, all the headers in Results, L343-345, L428, and several places in Discussion). I suggest doing a "find" for the word -transposition, and decide if that is truly accurate or should be replaced by "excision".

> This was done. The number of times transposition was used was reduced from 58 in the most recent version to 13 in the current version. “Transposition” was replaced by “excision” or “transposon excision” to reflect when excision was being measured.

A few other edits are here.
L80 have "been" identified

> This was done.

L93 with defective transposition (??) [not clear]

> This was changed and addressed in the editor’s comments of Line 114. The paragraph now reads:
>
> The T-DNA in the transformation vector, pEPS3004, contains the elements of the activation tagging sequences; i.) the non-autonomous transposon comprised of the 4X SCBV enhancer, the OsAct1p::AAD1 selectable marker and the terminal inverted repeats (TIR) from the Spm transposon and antibiotic marker, ii.) the transcription factors controlling anthocyanin biosynthesis, ZmGLOB1::B-peru and ZmGlob1::C1, and iii.) the Spm transposase.

L276-278 removes the disruption often leading to repair of the ......gene, and resulting in....

> This was changed to the reviewer’s recommendation. The sentence now reads:
>
> Excision of the non-autonomous transposable element removes the disruption and repairs the ZmGLOB1::B-peru gene resulting in anthocyanin production in tissues where the ZmGLOB1 promoter functions.

L285 (Fig. 2) not 3.

> This was done.

L285-287 embryos showed sectors of anthocyanin......suggesting that the transposable element excised, reconstituting a functional B-peru gene in these cells.

> This was done and now reads:
>
> …embryos showed sectors of anthocyanin accumulation suggesting that anthocyanin accumulation resulted in tissues where the transposable element is excised from the disrupted B-peru gene and repaired its structure to a functional B-peru gene in these cells.

L289 had excised [remove been]

This was done.

L294 collected from the purple sectors

This was done.

L296 in other studies characterizing transposon excision sites.

This was done

L324 (Fig. 4A), [remove period] 2) when excision occurs only in somatic tissues resulting in functional copies of the ....

This was done.

L374 with at least one purple [not on]

This was done.

L450 and DNA was isolated from seedling leaf tissue.

This was done.

L454 The transposon is not really a Spm but a dSpm, since it lacks transposase. Or just delete Spm and the sentence reads ok.

Spm was deleted.

L484 same as above

Spm was deleted.

L597 Nonetheless [one word]

This was changed.

L601-603 Could delete these first two sentences.

The first two sentences were deleted.
